# Analytical Models of Intra- and Extratumoral Cell Interactions at Avascular Stage of Growth in the Presence of Targeted Chemotherapy

**DOI:** 10.3390/bioengineering10030385

**Published:** 2023-03-21

**Authors:** Evgeniia Lavrenteva, Constantinos Theodoropoulos, Michael Binns

**Affiliations:** 1 Department of Chemical and Biochemical Engineering, Dongguk University-Seoul, 30 Pildong-ro 1-gil, Jung-gu, Seoul 04620, Republic of Korea; 2 Department of Chemical Engineering, Biochemical and Bioprocess Engineering Group, University of Manchester, Manchester M13 9PL, UK

**Keywords:** tumor growth model, computer modeling, avascular tumor, nutrient supply, chemotherapy, partial differential equations, tumor–immune interaction

## Abstract

In this study, we propose a set of nonlinear differential equations to model the dynamic growth of avascular stage tumors, considering nutrient supply from underlying tissue, innate immune response, contact inhibition of cell migration, and interactions with a chemotherapeutic agent. The model has been validated against available experimental data from the literature for tumor growth. We assume that the size of the modeled tumor is already detectable, and it represents all clinically observed existent cell populations; initial conditions are selected accordingly. Numerical results indicate that the tumor size and regression significantly depend on the strength of the host immune system. The effect of chemotherapy is investigated, not only within the malignancy, but also in terms of the responding immune cells and healthy tissue in the vicinity of a tumor.

## 1. Introduction

Cancerous malignancies are among the most lethal diseases affecting humanity over the last century. Moreover, they mutate and get more complex with time, which puts them at the top of the list of concerns for scientists and the medical society [1]. Conventional treatment techniques, such as chemotherapy, radiotherapy, immunotherapy, virotherapy, and surgical intervention, frequently do not lead to the full eradication of tumors [2]. Thus, there is an immense need for the precise theoretical prediction of the course of the disease under certain types of treatment and ways to make those therapies work in coordination, in order to achieve the most effective and tolerable results. The purpose of the mathematical modeling of cell interactions within tumors and affected tissues is to predict the effects of external factors, such as treatments, and to design or optimize these treatments (treatment dosages, treatment duration and combinations of different treatments) to minimize, stop or reverse tumor growth. 

Tumor-immune interactions and the effects of chemotherapeutic drugs are both of serious research interest, aiming to understand the dynamics of natural physiological responses to malignant formations and discover ways to use their benefits, along with therapeutic applications. To develop theoretical models that will closely represent in vivo conditions, many different quantitative approaches have been developed. 

The most basic models of tumor growth consider only general tumor cells as a formation. An example of this is the study of Song et al. [3], which was able to predict how tumor cells survive and evolve in terms of their encounters with the immune system, but does not account for other crucial phenomena, such as the effect of nutrition or the presence of differently behaving cell groups on the formation of this encounter, which could significantly alter the results. These types of models are typically represented by a small set of ordinary differential equations (ODEs). More sophisticated studies, still being general as regards the tumor dynamics, have also included the effect of the host immune system response and/or a treatment, leading to more realistic results, which fit well with experimental data [4,5]. Gupta et al. show how certain species of the immune system facilitate tumor destruction, using fractional order derivatives [4]. Elkaranshawy et al. includes the immune response and the effect of immunotherapy to make the realistic model [5]. These models extend the ODE set of equations by adding additional interaction terms.

A considerable number of authors has developed more complex descriptions and involved diverse cell populations into the model of a tumor [6,7]. Thus, Sherratt and Chaplain formulated a new extended model that consists of all cell types present in clinically observed tumor samples, and take into account the role of nutrition received from the underlying tissue distributed among live cells [6]. Taghibakhshi et.al. investigated the effects of the concentrations of the essential nutrients and of the initial spheroid radius on the tumor growth [7]. These models essentially describe diffusion processes and are given as sets of partial differential equations (PDEs).

Separately, a large body of research has looked at the effect of chemotherapeutic drugs on tumor growth and regression. As an example, Ansarizadeh et al. [8] suggest a complex model, which describes the interaction among normal, immune and tumor cells in a tumor with a chemotherapeutic drug, using a set of coupled PDEs. Moreover, there are approaches such as that in [9], designed by Pourhasanzade et al., which even look into the microenvironmental factors, as well as at separate types of nutrients and various cell groups of a tumor. These types of models appear to be more detailed and sophisticated PDE sets.

As can be seen from Table 1, different models have been developed for the prediction of tumor growth, considering the effects of different factors employing a range of assumptions. For example, many studies consider a single type of generic tumor cells [3,4,5,8,10,11,12,13,14,15], while others consider separate proliferating, quiescent and necrotic cells within the tumor [6,7,9,16,17,18]. The benefit of considering these separate types of cells is a full understanding of intra-tumoral interactions, which prove to be more realistic than a generalized approach. 

Nowadays, a clear picture of a tumor can hardly be complete and competent in its applications, if the major latest findings and clinical observations are neglected. Thus, to produce more realistic model predictions and to aid the development of treatments, this study develops a model based on the one in [6], including a larger number of factors than previous studies, leading to a more complex, but more useful, model, which can account for various interactions between different cell types, as well as for interactions with nutrient levels, immune response and chemotherapeutic treatment. A few of the previous models similarly based on the model in [6] attempted to consider certain aspects of tumor growth, but they often lacked one or another crucial parameter, or used some degree of generalization, in order for the model to be realistic compared to physiological observations.

This paper is divided into four sections: in Section 2, we present a formulation of the mathematical model governed by a partial differential equations system, and the description of its parameters, modifications, and extensions, developed to include innate immunity response, chemotherapy, and glucose/oxygen consumption. Section 3 delves into the model's responses to varying conditions of the tumor microenvironment and immune-chemotherapy treatment strategies, while also conducting model validation against existing experimental results. Our assessment of the computational results and graphical data obtained by altering model parameters considers potential biological outcomes and offers optimal treatment predictions. Finally, Section 4 concludes the article with a summary of our findings and directions for future work.

## 2. Mathematical Model and Methodology

In this segment, we have developed and verified a new, complex, extended model of an avascular tumor formation, representing the interactions and mutual relations of intratumoral cell populations—proliferating cells (PC), quiescent cells (QC), necrotic cells (NC)—with extratumoral cell populations—surrounding healthy tissue cells (SC) and immune system cells, cytotoxic to the active (PC and QC) cancerous cells (IC)—as well as nutrient consumption and chemotherapeutic drug intake over time.

By considering the formation of cancer as one cluster of atypical cells or neglecting its interaction with other tissues in contiguity, gives an opportunity of constructing a simpler model, but apparently it does not illustrate the process to the fullest extent. Therefore, we suggest a system that takes cell species inside and outside of the tumor, as well as the presence of nutrients, under chemotherapy’s effect, into account. The model parameters are estimated using experimental data, and reflect clearly how the proposed PDE system describes the system dynamics and predicts tumor growth. Sensitivity analysis of the system provides the characterization of a significantly improved microenvironment, and therapeutic conditions needed for a tumor to shrink in the smallest time interval.

A numerical model was developed, with the aim of investigating the interactions between the participating intra-tumoral and extra-tumoral cell populations. This model considers proliferating, quiescent and necrotic cells, in addition to healthy host cells and generic immune response cells. Additionally, the model accounts for nutrient sufficiency and the individual immune response strength, as well as the effect of a chemotherapeutic medicament on avascular stage cancer tumor growth. 

The model is constructed as a set of seven partial differential equations, and it is well-defined in terms of cell densities of proliferating (living) cells *P(x,t)*, quiescent (nonproliferating live) cells *Q(x,t)*, necrotic cells *N(x,t)*, surrounding cells *S(x,t)*, attacking immune cells *L*, nutrient supply influx *C(x,t)* and a chemotherapeutic drug influx *D(x,t)*. The chemotherapy schedule is assumed to be the same as in the work of [8].

### 2.1. Model Assumptions

The proposed model is based on the following assumptions:

- Chemotherapy and innate immune responses decrease proliferation.
- All living cells receive nutrients (consisting of glucose and oxygen) from the underlying tissue, and divide depending on the level of nutrient supply.
- Chemotherapeutic drugs attack proliferating cells, surrounding healthy cells and immune cells.
- One cell population limits the movement of the cell population of another type and vice versa—this phenomenon is called ‘contact inhibition of migration’ [6].
- The effectiveness of the nutrient source term decreases with overall cell density.
- We assume that the nutrients, the immune response, and the drug react, and diffuse over the spatial domain.
- Nutrients diffuse into the tumor space at a diffusion rate that allows for the concentration of nutrient supply to reach a steady state.
- Immune cells are generated through a steady influx into the tumor area, and proliferate within it.

### 2.2. Modelling Equations

The cell density, *P*, dynamics of the proliferating rim can be described as a diffusion equation:

(1) $$\frac{\partial P}{\partial t}=\frac{\partial }{\partial x}\left[\frac{P}{P+Q+S}\frac{\partial }{\partial x}\left(P+Q+S\right)\right]+g\left(C\right)P\left(1-P-Q-N-S\right)-f\left(C\right)P-k_{5}LP-k_{DP}\left(1-e^{-D}\right)P$$

The cell density, *Q*, of the quiescent cell population is given by

(2) $$\frac{\partial Q}{\partial t}=\frac{\partial }{\partial x}\left[\frac{Q}{P+Q+S}\frac{\partial }{\partial x}\left(P+Q+S\right)\right]+f\left(C\right)P-h\left(C\right)Q-k_{DQ}\left(1-e^{-D}\right)Q$$

and cell density, *N*, of the necrotic core by :

(3) $$\frac{\partial N}{\partial t}=h\left(C\right)Q$$

The cell density of healthy tissue surrounding the tumor, *S*, dynamics is described by:

(4) $$\frac{\partial S}{\partial t}=\frac{\partial }{\partial x}\left[\frac{S}{P+Q+S}\frac{\partial }{\partial x}\left(P+Q+S\right)\right]+g\left(C\right)S\left(\Gamma -P-Q-N-S\right)-k_{DS}\left(1-e^{-D}\right)S$$

The nutrient supply from the underlying tissue, *C*, is given by:

(5) $$\frac{\partial C}{\partial t}=D_{c}\frac{\partial^{2}C}{\partial x^{2}}+k_{1}C_{0}\left[1-\alpha \left(P+Q+N+S\right)\right]-k_{1}C-k_{2}PC-k_{3}SC-k_{4}LC$$

The response of the innate immune system, *L*, dynamics can be computed as:

(6) $$\frac{\partial L}{\partial t}=D_{L}\frac{\partial^{2}L}{\partial x^{2}}-v_{L}\frac{\partial L}{\partial x}+g(C)L\left(\Gamma -P-Q-N-S-L\right)-k_{5}PL-k_{DL}(1-e^{-D})L$$

The chemotherapy drug intake, *D*, is as follows:

(7) $$\frac{\partial D}{\partial t}=D_{D}\frac{\partial^{2}D}{\partial x^{2}}+v_{D}(t)-k_{D}D$$

Here 

$k_{DP}(1-e^{-D})P$, $k_{DQ}(1-e^{-D})Q$, $k_{DS}(1-e^{-D})S$, and$k_{DL}(1-e^{-D})L$ indicate the fractional cell kill rate, and term $\left(1-e^{-D}\right)$ represents saturation. For each type of cell, the fractional cell kill rates under the chemotherapy effect are denoted by $k_{DP}$, $k_{DQ}$, $k_{DS}$ and $k_{DL}$, for proliferating, quiescent, healthy surrounding, and immune cells, respectively. The source term $k_{1}C_{0}\left[1-\alpha \left(P+Q+N+S\right)\right]$ in Equation (5) represents the access of nutrition from the surrounding tissue and $C_{0}$ is the nutrient concentration in the absence of a tumor cell population. The term $-k_{1}C$ describes the nutrient natural decay, whereas $-k_{2}PC$, $-k_{3}SC$, and $-k_{4}LC$ define the nutrient consumption by proliferating, surrounding and immune cells, respectively. In Equations (1)–(3) and (6), the functional form h(C) denotes the rate of quiescent cells turning to necrotic ones, g(C) is the mitosis rate of proliferating cells, whereas f(C) is the rate of proliferating cells turning quiescent. Furthermore, f(C) and h(C) are decreasing functions, whereas function g(C) is an increasing function. These functional forms are selected with parameter values g(C)=1+0.2C, h(C)=0.5f(C), f(C)=0.5[tanh(4C−2)]. Γ and α are dimensionless parameters, Γ<1 and $\alpha \in \left[0,1\right]$. Equations (5)–(7) introduce the diffusive terms $D_{C}\frac{\partial^{2}C}{\partial x^{2}}$, $D_{L}\frac{\partial^{2}L}{\partial x^{2}}$, and $D_{D}\frac{\partial^{2}D}{\partial x^{2}}$ into the system, where diffusion coefficients for nutrients, immune system cells and the chemotherapeutic drug are represented by $D_{C}$, $D_{L}$ and

$D_{D}$, respectively.

The external pulsing source of the chemotherapeutic drug $v_{D}(t)$ in Equation (7) is denoted as the dosage strength:

$$v_{D}(t)=1for\;\left(n-1\right)\times i<t<\left(n-1\right)\times i+\tau ,\;else\;0,$$

where i = 7 days is the interval, τ = 0.25 days is the dosage duration and the number of pulses is taken as n = 1, 2, 3, according to the work of Ansarizadeh et al. [8]. Additionally, the term $-k_{D}D$ represents the excretion of the drug.

The initial conditions for our model are used in accordance with the original model [6]:

$$\begin{array}{c} P\left(x,0\right)=0.125e^{-0}{}^{.1x},Q\left(x,0\right)=0,N\left(x,0\right)=0,\\ S\left(x,0\right)=\Gamma \times (1-0.01e^{-0}{}^{.1x}),L\left(x,0\right)=1,C=1,D=1 \end{array}$$.

The boundary conditions for x=0 and x=265 are assumed as follows:

$$\frac{\partial P}{\partial x}=0,\frac{\partial Q}{\partial x}=0,\frac{\partial S}{\partial x}=0,\frac{\partial L}{\partial x}=0,\frac{\partial C}{\partial x}=0,\frac{\partial D}{\partial x}=0$$

where for x= 0, these conditions represent symmetry, while for x = 265, the selected simulated boundary, they represent a “flat” profile. The original model this is based on does not mention the units used [6], but through comparison with other studies such as [8], it can be determined that 100 units of x equate to 1 cm, and the units of time are days.

In its turn, N requires no boundary condition. In order to compute a solution for the system described in Equations (1)–(7), the space-step and time-step were selected, obtaining the values of Δx = 0.5 and Δt = 0.05, respectively. The model’s parameters are given in Table 2.

## 3. Model Results and Validation

This section explains the model results for five different cell populations, a set of parameters and a range of initial and boundary conditions. The numerical simulations were conducted by employing the finite difference method (FDM), as it is robust and simple for solving partial differential equations, where the solution region consists of regular geometries. To determine the dynamics behavior of the system, an explicit Euler method was employed. Although more sophisticated numerical integration methods, such as those employing variable step length and implicit methods exist, for simplicity, the simpler, explicit Euler method was used here. If a larger time-step is used, this can lead to inaccuracies, so different time steps were tested, and a small enough time-step was used, such that any integration error was negligible.

### 3.1. Chemotherapy Effect on Malignant Proliferation and Tumor Regression

**Figure 1:**
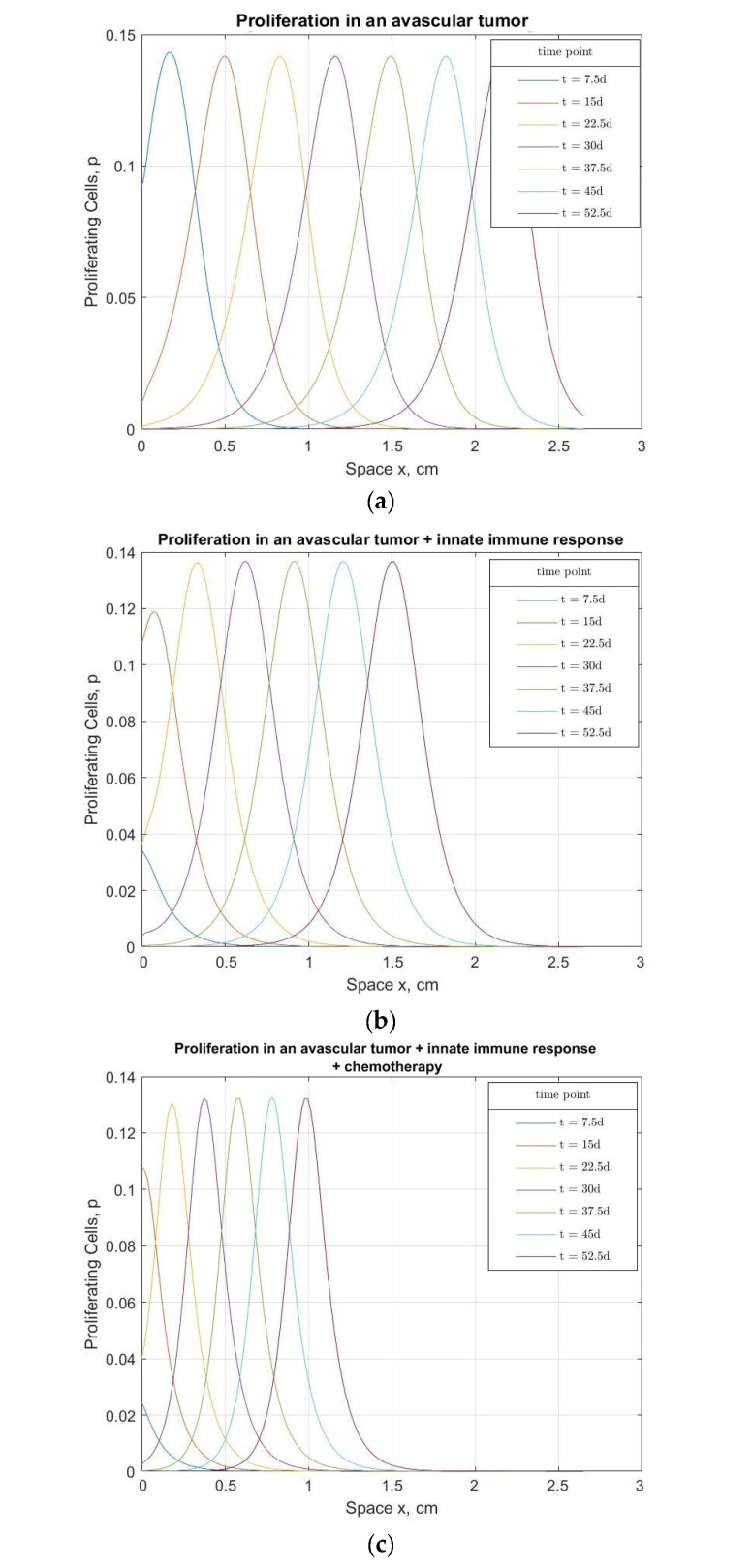
Numerical solutions for the proliferating cell density of an avascular tumor (**a**) without extratumoral interactions, (**b**) with the response of the innate immune system, (**c**) with the response of the innate immune system under the effect of chemotherapy, as functions of space x at times t = 0…52.5 days.

In the present model, the growth of a tumor was evaluated by the density of the proliferating cell population P, which is an indicator of malignancy. Figure 1 displays that the proliferating cell density decreases when the innate immune system is involved (b) and, logically, decreases even more in the presence of chemotherapy (c); these results suggest that the model gives the expected response trends. In this study, the chemotherapeutic drug was assumed to be an agent with the parameters presented in the previous related works [8,22,23], and does not represent a specific drug. The model is supposed to give the opportunity to fit and estimate the drug parameters, so the particular medication can be chosen according to its kinetic features.

The cell density in the basic model goes up to the value of 0.14 × 10^9^ cells/cm^3^, a point at which the immune cells affect the proliferation rate, while density slightly decreases. In turn, the drug intake results in a decrease of density to 0.13 × 10^9^ cells/cm^3^. We assume that, in all cases, the nutrient supply *C* is accessed by the proliferating rim of the tumor from the underlying tissue, and functionally regulates the ability of proliferating cells to enter quiescence; the nutrient coefficient α value tends to be relatively small. It can clearly be seen that the incorporation of the diffusive terms $\frac{\partial L}{\partial t}$ and consequently $\frac{\partial D}{\partial t}$, for the immune response and the chemotherapy effect respectively, provides a realistic representation of tumor growth and regression dynamics.

**Figure 2:**
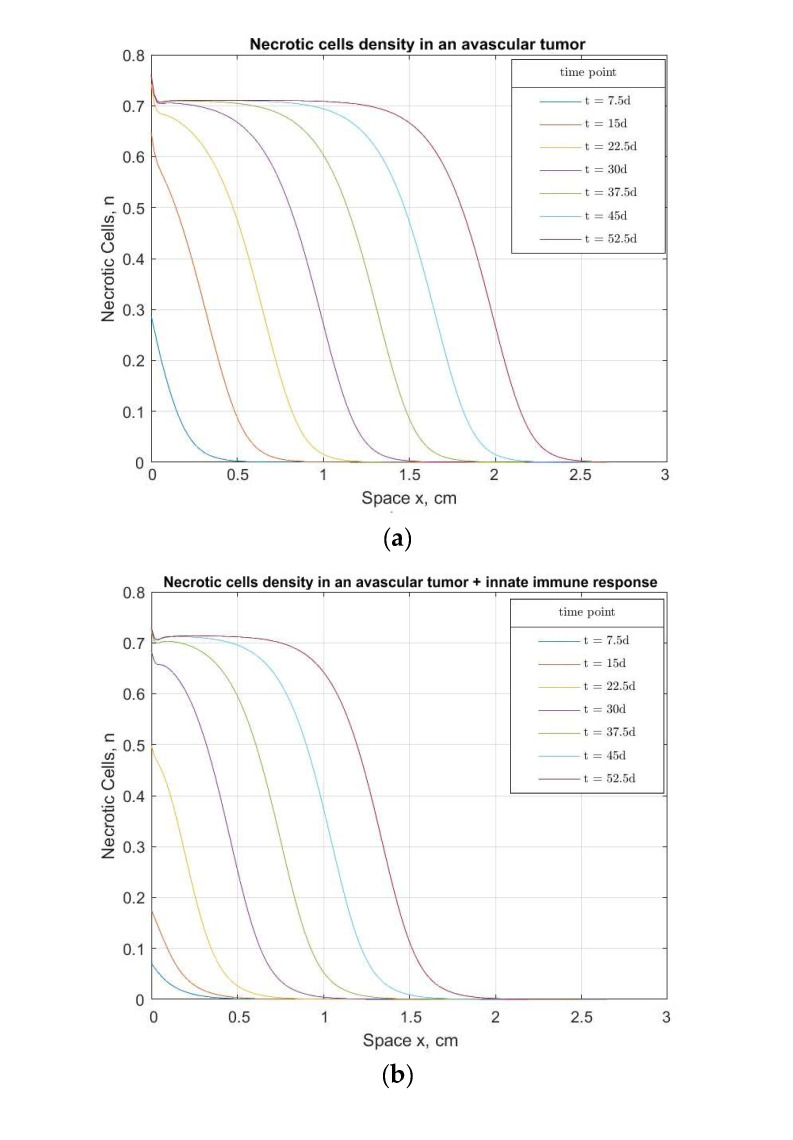

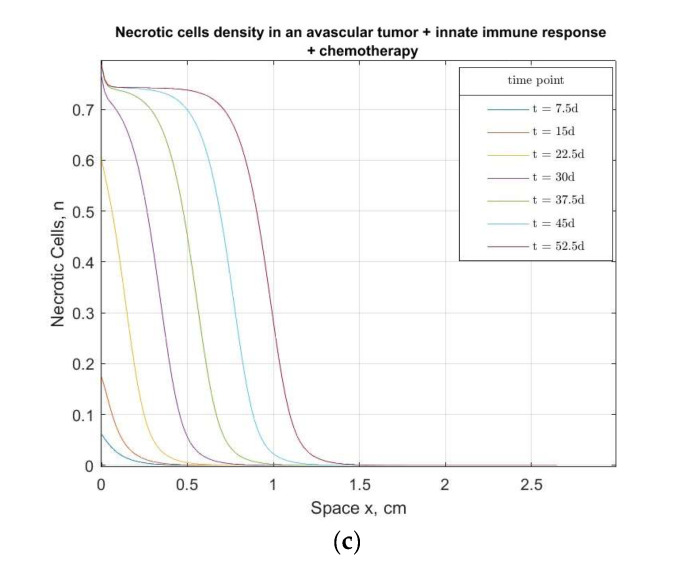
Numerical solutions for the necrotic cell density of an avascular tumor (**a**) without extratumoral interactions, (**b**) with the response of the innate immune system, (**c**) with the response of the innate immune system under the effect of chemotherapy, as functions of space x at times t = 0…52.5 days.

In Figure 2, the necrotic cell density *N* is presented for all three considered steps of the model. Figure 2a shows the density of a simple avascular formation without any side effects, yielding approximately 0.7 × 10^9^ cells/cm^3^ (after 30 days). With the addition of the immune system cells’ effects in Figure 2b, the number of necrotic cells stops growing, meaning that the number of quiescent turning necrotic and, therefore, the number of proliferating cells turning quiescent, has declined as well, to 0.69 × 10^9^ cells/cm^3^. After the introduction of a chemotherapeutic drug in Figure 2c, the necrotic cell density slightly decreases, which supports the earlier finding in Figure 1c, regarding the decrease in proliferation.

### 3.2. The Immunity Power Effect on Tumor Evolvement under Chemotherapy

**Figure 3:**
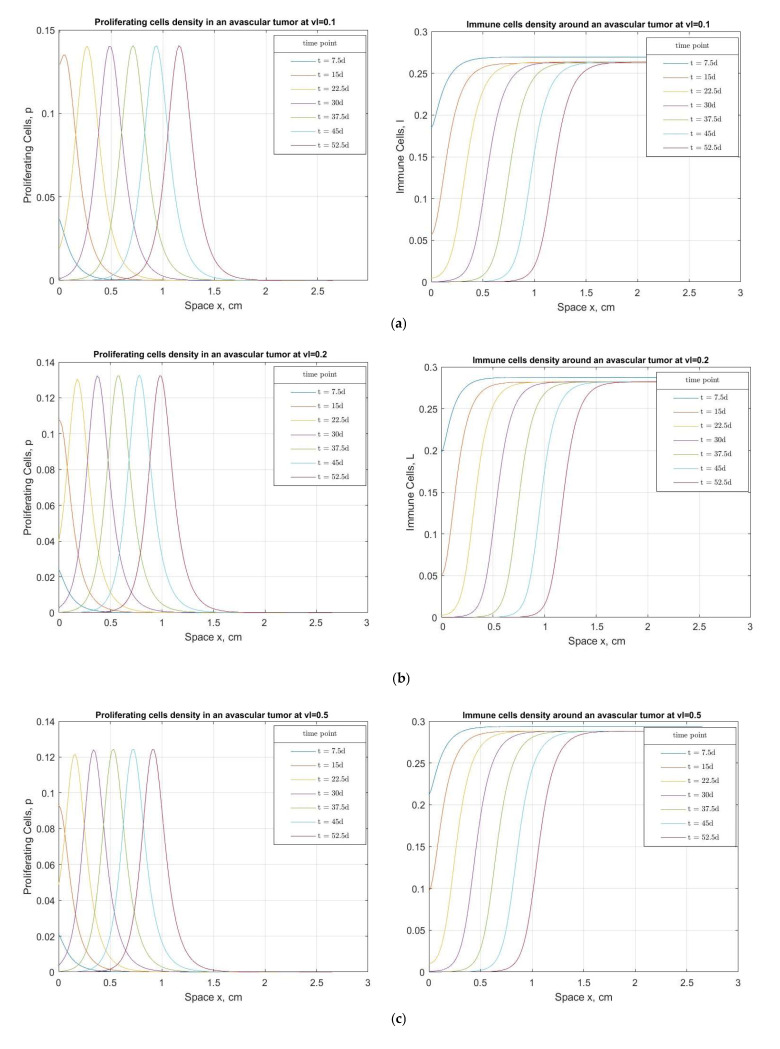
Numerical solutions of proliferating and immune cell densities, for three different levels of immune responses at (**a**) $v_{L1}$ = 0.1, (**b**) $v_{L2}$ = 0.2, (**c**) $v_{L3}\;$= 0.5 under the effect of chemotherapy, as functions of space x at times t = 0…52.5 days.

We have considered three different levels of immune response, varying the rate of the external immune cells influx from the medium value$\;v_{L2}$ = 0.2, used in recent relevant works, and testing sensitivities with low and high values: $v_{L1}$ = 0.1 and $v_{L3}\;$ = 0.5. Figure 3 shows that patients with different immune system power experience different effects on tumor formation under the treatment of the same drug. The graph suggests that malignant proliferation is faster when the immune system response is weaker (*P* ≈ 0.143 × 10^9^ cells/cm^3^, $v_{L1}$ = 0.1, after 30 days). Otherwise, a patient with a stronger immune response has a decline in proliferation speed (*P* ≈ 0.123 × 10^9^ cells/cm^3^, $v_{L3}$ = 0.5, after 30 days). The model results are consistent with clinical findings that treatment is more effective in cases where a stronger innate immune system response is present. Therefore, we suggest that immunotherapy, which boosts the immune response, should be combined with other treatments and models, such as the one presented in this study, which should be used to determine the optimal combination of immunotherapy and other treatments.

### 3.3. Chemotherapy’s Effect on the Surrounding Healthy Tissue and Immune System

**Figure 4:**
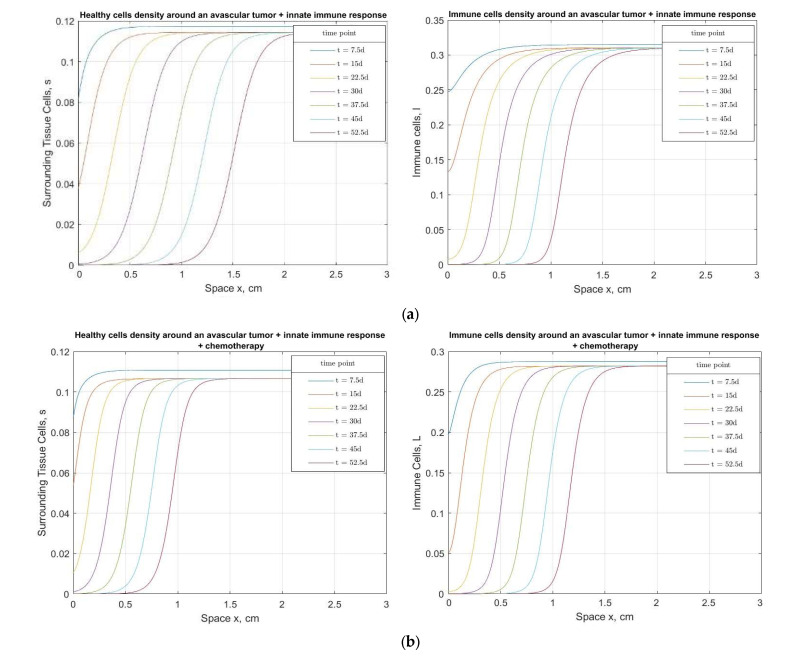
Numerical solutions of surrounding healthy and immune cell densities (**a**) before the drug intake, (**b**) under the effect of chemotherapy, as functions of space x at times t = 0…52.5 days.

Chemotherapy medications cannot differentiate between tumor cells and healthy cells. This is the reason why chemotherapy damages healthy cells, lowers immune cell counts and causes other negative side effects. The model output presented in Figure 4 demonstrates the influence of a chemotherapeutic drug on cells that are healthy, and preferably should not be affected be the treatment; in the present case the drug promotes considerable changes in the non-malignant cell populations of this model.

In Figure 4a the graphs show cell densities’ progression over time before the implementation of a chemotherapeutic drug, with the surrounding tissue cells S and the immune system cells L. Meanwhile, Figure 4b provides the graphs of the same terms under the effect of chemotherapy. It is obvious from the solution that the drug affects both healthy cell species and tumor cells, as denoted by the decreases in their densities.

### 3.4. Model Validation

Our model was compared for prediction accuracy with the available models that describe the tumor growth in terms of cell densities (Table 3). The obtained data were also compared against experimental data, as well as other modeling results. For this purpose, the model of Swanson et al. [24] was chosen, as it presents results that quantitatively describe the dynamics of avascular aggressive tumor formation, visualized by the medical imaging of multiple patients. Next, the model of Hinow et al. [25] allows us to compare the tumor (proliferating) cell densities after chemotherapy treatment was carried out. Another piece of evidence that helps us validate some terms of the new model is an experiment of Chicoine and Silbergeld [26]; this was an in vitro tumor cell assay cultivated in a Petri dish. Here, the medium cell density of the tumor cells is considered.

The absence of models with the same number and type of variables makes it possible to validate several terms present in the developed model, but not all. However, the results (cell density profiles) match those given in the original model of Sherratt and Chaplain [6] if the extra terms added here are neglected, which gives us the confidence to assume that our model shows the right trends.

The work of Wang et al. [27] shows that the normalized drug concentration in our model tends to have a stable, constant distribution rate, similar to their findings. Additionally, [27] is supported by solid experimental validation. The immune response rate can be validated through the article of Ku-Carillo et al. [28], where the immune cell density for the low-level immune strength ($v_{L1}$ = 0.1) appears to be *I* ≈ 0.4 × 10^9^ cells/cm^3^, whereas for the case of an intermediate level of immune strength, *I* significantly increases. In a similar way, this model, in terms of the immune system response, results in *I* ≈ 0.26 × 10^9^ cells/cm^3^ ($v_{L1}$ = 0.1), which increases with the increase in immune strength.

Future work should involve increasing the model’s complexity and adding more supportive experimental validation, in order to prove its potential, in terms of its application to certain types of cancer.

## 4. Conclusions

We constructed an extended PDE-based model to represent the interactions between intra- and extratumoral cell populations limited by nutrition level and chemotherapeutic drug intake. The proposed model has been created based on an earlier developed model [6], which was extended by incorporating the effects of immune response and chemotherapy, including their coupling/interactions and using avascular tumor growth experimental data. Having reviewed a number of existing models, we suggest a new model, which integrates all relevant terms into one larger system that is equally realistic and practically applicable. We tested the performance of the designed model through numerical calculations, which match the literature, as well as experimental data shown in the works of Swanson et al. [24], Hinow et al. [25], Chicoine and Silbergeld [26]. The set of equations was implemented by using a finite difference method in MatLab.

According to the simulation results, in the created interactive system, a tumor’s progression drastically depends on the following: (a) a patient’s innate immunity level—a powerful immune response significantly changes the proliferation rate; (b) the nutrient supply—the limitations in nutrition obviously affect the proliferating cells’ spread but also the immune response and the healthy tissue around the formation. These findings establish our model’s accuracy, adequacy and effectiveness. The graphical depiction demonstrates logical trends for cell population interactions, proliferation and death. In view of these recounted facts, we suggest that the proposed model is suitable for predicting the distribution and behavior of the different cell types in this complex multicellular formation. In conclusion, the constructed model can be improved and utilized to further examine the malignant processes in the damaged tissue on early stages, the sensitivity of the drug diffusion and decay, nutrient supply and the level and duration of chemotherapy, in order to suggest the most optimal treatment. In our future research, we plan to incorporate other factors, such as radiotherapy and/or immunotherapy, in order to simulate and analyze the most-effective combined treatment strategies.

## Figures and Tables

**Table 1:** Recent mathematical models of tumor growth.

Parameter	Generalized Tumor Cells	Cell Populations of a Tumor			Surrounding Healthy Tissue Cells	Innate Immune Cells (Varied)	Chemotherapeutic Drug Effect	Nutrients	Immunotherapy Effect	Other
Author		Proliferating Cells	Quiescent Cells	Necrotic Cells						
This study		⁕	⁕	⁕	⁕	⁕	⁕	⁕		
[4]	⁕					⁕	⁕			
[19]		⁕	⁕					⁕		
[17]		⁕	⁕	⁕	⁕			⁕		
[7]		⁕	⁕	⁕				⁕		
[9]		⁕	⁕	⁕	⁕			⁕		
[12]	⁕					⁕	⁕			
[3]	⁕					⁕				
[18]		⁕	⁕	⁕				⁕		⁕
[16]		⁕		⁕		⁕				
[5]	⁕					⁕	⁕		⁕	
[20]	⁕						⁕			⁕
[14]	⁕					⁕	⁕		⁕	
[6]		⁕	⁕	⁕	⁕			⁕		
[10]	⁕					⁕	⁕		⁕	
[11]	⁕					⁕			⁕	
[13]	⁕					⁕	⁕		⁕	
[15]	⁕					⁕	⁕			
[21]		⁕		⁕		⁕				
[8]	⁕				⁕	⁕	⁕			

⁕ Parameter of an avascular tumor mathematical model considered in a research paper.

**Table 2:** Model parameters.

Parameter	Value	Unit	Reference	Description
$k_{DP}$	0.3	${\mathrm{days}}^{-1}$	[8]	Fraction cell kill rate of proliferating cells under chemotherapeutic drug effect (value used for generalized tumor cells)
$k_{DQ}$	0.1	${\mathrm{days}}^{-1}$	[8]	Fraction cell kill rate of quiescent cells under chemotherapeutic drug effect (assumed the same as for healthy cells)
$k_{DS}$	0.1	${\mathrm{days}}^{-1}$	[8]	Fraction cell kill rate of healthy surrounding tissue cells under chemotherapeutic drug effect
$k_{DL}$	0.2	${\mathrm{days}}^{-1}$	[8]	Fraction cell kill rate of immune system cells under chemotherapeutic drug effect
$D_{C}$	10	${\left(100\;\mu m\right)}^{2}\;{\mathrm{days}}^{-1}$	[6]	Diffusion coefficient of the nutrient
$D_{L}$	5	${\left(100\;\mu m\right)}^{2}{\;\mathrm{days}}^{-1}$	estimated	Diffusion coefficient of the immune system cells
$D_{D}$	8	${\left(100\;\mu m\right)}^{2}{\;\mathrm{days}}^{-1}$	estimated	Diffusion coefficient of a chemotherapeutic drug
$C_{0}$	1	dimensionless	[6]	Nutrient concentration in the absence of abnormal proliferation
$v_{L}$	0.2	${\mathrm{days}}^{-1}$	[22]	Rate of the external immune cell influx
$k_{1}$	8	${\mathrm{days}}^{-1}$	[6]	Decay rate of the nutrient
$k_{2}$	1	${\mathrm{days}}^{-1}$	[6]	Rate of the nutrient consumption by proliferating cells
$k_{3}$	1	${\mathrm{days}}^{-1}$	[6]	Rate of the nutrient consumption by quiescent cells
$k_{4}$	1	${\mathrm{days}}^{-1}$	[8]	Rate of immune cells death after contacting proliferating cells
$k_{5}$	0.55	${\mathrm{days}}^{-1}$	[8]	Rate of proliferating cells death after contacting immune cells
$k_{6}$	1	${\mathrm{days}}^{-1}$	estimated	Rate of the nutrient consumption by immune cells
$k_{D}$	0.2	${\mathrm{days}}^{-1}$	[23]	Decay rate of a chemotherapeutic drug
*α*	0.8	dimensionless	[6]	Nutrient coefficient
*Γ*	0.4	dimensionless	[6]	Dimensionless parameter

**Table 3:** Validation of the proposed model.

Parameter	Present Model	Lit. Model [24]	Lit. Model [25]	Experiment [26]
Density of proliferating cells (before chemotherapy)	~0.145 × 10^9^ cells/cm^3^	~0.20 × 10^9^ cells/cm^3^ (normoxic tumor cells)	~	~0.15 × 10^9^ cells/cm^3^ (normalized density of cancer cells)
Density of necrotic cells	~0.75 × 10^9^ cells/cm^3^	~0.95 × 10^9^ cells/cm^3^ (necrotic tissue)	~	~
Density of proliferating cells (after chemotherapy)	~0.13 × 10^9^ cells/cm^3^	~	~0.1 × 10^9^ cells/cm^3^ (normoxic tumor cells)	~

## Data Availability

Data is contained within the article and in references. Parameter values and experimental data used for estimating parameters and validation can be found in references [6,22,23,24,25,26,27,28]. Parameters used in this study can be found in Table 2 in this article. Matlab codes for the different models can be found in the Supplementary Materials.

## Supplementary Materials

The following supporting information can be downloaded at: https://www.mdpi.com/article/10.3390/bioengineering10030385/s1. Three sets of Matlab codes used to obtain the results obtained here: A basic model, A model which additionally includes immune response, A model which additionally includes immune response and chemotherapy.

## Nomenclature

*P*	Proliferating cell population density, (×10^9^ to give units cells/cm^3^)
*Q*	Quiescent cell population density, (×10^9^ to give units cells/cm^3^)
*N*	Necrotic cell population density, (×10^9^ to give units cells/cm^3^)
*S*	Surrounding tissue cell population density, (×10^9^ to give units cells/cm^3^)
*C*	Nutrient supply intake
*L*	Immune cell population density, (×10^9^ to give units cells/cm^3^)
*D*	Chemotherapy drug intake
*k_DP_*	$\mathrm{Fraction}\;\mathrm{cell}\;\mathrm{kill}\;\mathrm{rate}\;\mathrm{of}\;\mathrm{proliferating}\;\mathrm{cells}\;\mathrm{under}\;\mathrm{chemotherapeutic}\;\mathrm{drug}\;\mathrm{effect}\;\left({\mathrm{days}}^{-1}\right)$
*k_DQ_*	$\mathrm{Fraction}\;\mathrm{cell}\;\mathrm{kill}\;\mathrm{rate}\;\mathrm{of}\;\mathrm{quiescent}\;\mathrm{cells}\;\mathrm{under}\;\mathrm{chemotherapeutic}\;\mathrm{drug}\;\mathrm{effect}\;\left({\mathrm{days}}^{-1}\right)$
*k_DS_*	$\mathrm{Fraction}\;\mathrm{cell}\;\mathrm{kill}\;\mathrm{rate}\;\mathrm{of}\;\mathrm{surrounding}\;\mathrm{tissue}\;\mathrm{cells}\;\mathrm{under}\;\mathrm{chemotherapeutic}\;\mathrm{drug}\;\mathrm{effect}\;\left({\mathrm{days}}^{-1}\right)$
*k_DL_*	$\mathrm{Fraction}\;\mathrm{cell}\;\mathrm{kill}\;\mathrm{rate}\;\mathrm{of}\;\mathrm{immune}\;\mathrm{system}\;\mathrm{cells}\;\mathrm{under}\;\mathrm{chemotherapeutic}\;\mathrm{drug}\;\mathrm{effect}\;\left({\mathrm{days}}^{-1}\right)$
*D_C_*	$\mathrm{Diffusion}\;\mathrm{coefficient}\;\mathrm{of}\;\mathrm{the}\;\mathrm{nutrient}\;\left({\left(100\;\mu m\right)}^{2}{\mathrm{days}}^{-1}\right)$
*D_L_*	$\mathrm{Diffusion}\;\mathrm{coefficient}\;\mathrm{of}\;\mathrm{the}\;\mathrm{immune}\;\mathrm{system}\;\mathrm{cells}\;\left({\left(100\;\mu m\right)}^{2}{\mathrm{days}}^{-1}\right)$
*D_D_*	$\mathrm{Diffusion}\;\mathrm{coefficient}\;\mathrm{of}\;a\;\mathrm{chemotherapeutic}\;\mathrm{drug}\;\left({\left(100\;\mu m\right)}^{2}{\mathrm{days}}^{-1}\right)$
*C_0_*	Nutrient concentration in the absence of abnormal proliferation
v_L_	$\mathrm{Rate}\;\mathrm{of}\;\mathrm{the}\;\mathrm{external}\;\mathrm{immune}\;\mathrm{cells}\;\mathrm{influx}\;\left({\mathrm{days}}^{-1}\right)$
*k_1_*	$\mathrm{Decay}\;\mathrm{rate}\;\mathrm{of}\;\mathrm{the}\;\mathrm{nutrient}\;\left({\mathrm{days}}^{-1}\right)$
*k_2_*	$\mathrm{Rate}\;\mathrm{of}\;\mathrm{the}\;\mathrm{nutrient}\;\mathrm{consumption}\;\mathrm{by}\;\mathrm{proliferating}\;\mathrm{cells}\;\left({\mathrm{days}}^{-1}\right)$
*k_3_*	$\mathrm{Rate}\;\mathrm{of}\;\mathrm{the}\;\mathrm{nutrient}\;\mathrm{consumption}\;\mathrm{by}\;\mathrm{quiescent}\;\mathrm{cells}\;\left({\mathrm{days}}^{-1}\right)$
*k_4_*	$\mathrm{Rate}\;\mathrm{of}\;\mathrm{immune}\;\mathrm{cells}\;\mathrm{death}\;\mathrm{after}\;\mathrm{contacting}\;\mathrm{proliferating}\;\mathrm{cells}\;\left({\mathrm{days}}^{-1}\right)$
*k_5_*	$\mathrm{Rate}\;\mathrm{of}\;\mathrm{proliferating}\;\mathrm{cells}\;\mathrm{death}\;\mathrm{after}\;\mathrm{contacting}\;\mathrm{immune}\;\mathrm{cells}\;\left({\mathrm{days}}^{-1}\right)$
*k_6_*	$\mathrm{Rate}\;\mathrm{of}\;\mathrm{the}\;\mathrm{nutrient}\;\mathrm{consumption}\;\mathrm{by}\;\mathrm{immune}\;\mathrm{cells}\;\left({\mathrm{days}}^{-1}\right)$
*k_D_*	$\mathrm{Decay}\;\mathrm{rate}\;\mathrm{of}\;a\;\mathrm{chemotherapeutic}\;\mathrm{drug}\;\left({\mathrm{days}}^{-1}\right)$
*α*	Nutrient coefficient
*Γ*	Dimensionless parameter in Equation (4)
*f*	Functional form representing the rate of quiescent cells turning to necrotic
*h*	Functional form representing the rate of proliferating cells turning quiescent
*g*	Functional form representing the mitosis rate of proliferating cells
*v_D_(t)*	$\mathrm{External}\;\mathrm{pulsing}\;\mathrm{source}\;\mathrm{of}\;\mathrm{the}\;\mathrm{chemotherapeutic}\;\mathrm{drug}\;\left({\mathrm{days}}^{-1}\right)$
